# The Study of Effectiveness of Blended Learning Approach for Medical Training Courses

**Published:** 2012-01-01

**Authors:** Z Karamizadeh, N Zarifsanayei, A A Faghihi, H Mohammadi, M Habibi

**Affiliations:** 1 Department of Pediatric Endocrine, Shiraz University of Medical Sciences, Shiraz, Iran; 2 Distance Educational Planning Center of Excellence for e-Learning in Medical Sciences, Shiraz, Iran; 3 Research Scholar in Education, Shiraz University of Medical Sciences, Shiraz, Iran; 4 Department of Pediatrics, Shiraz University of Medical Sciences, Shiraz, Iran; 5 Department of Medical Education, Shiraz University of Medical Sciences, Shiraz, Iran

**Keywords:** Blended Learning, Efficacy, Medical Students

## Abstract

**Background:**

Blended learning as a method of learning that includes face to face learning, pure E-learning and didactic learning. This study aims to investigate the efficacy of medical education by this approach.

**Methods:**

This interventional study was performed in 130 students at different clinical levels participating in class sessions on "congenital adrenal hyperplasia and ambiguous genitalia". Sampling was done gradually during 6 months and all of them filled a pretest questionnaire and received an educational compact disk. One week later, a presence class session was held in a question and answer and problem solving method. Two to four weeks later, they filled a posttest questionnaire.

**Results:**

There was a significant correlation between pretest and posttest scores and the posttest scores were significantly more than the pretest ones. Sub-specialized residents had the most and the students had the least attitude towards blended learning approach. There was a significant correlation between the research samples' accessibility to computer and their attitude and satisfaction to blended learning approach.

**Conclusion:**

Findings generally showed that the blended learning was an effective approach in making a profound learning of academic subjects.

## Introduction

Blended learning as a method of learning that includes face to face learning, pure E-learning and didactic learning. It aims to improve the quality and develop the quantity of educational activities in two vertical and horizontal dimensions by the use of different instruments and progressive technologies. In the horizontal dimension, it tries to expand the extent of instruments which facilitate learning in a learning strategy, in such a way that with their best combination, the highest quality would be obtained. In the vertical dimension, it proceeds to the in-depth analysis of learning and the better understanding of educational material, and what techniques at what times led to a better understanding of the subjects and optimized learning.[1][2][3][4]

Medical education is one of the fields that blended learning is effective in. Blended education can fill the gap between theory and practice and it can encourage the learner to solve problems and exchange experiences.[3][4][5] According to the mentioned reasons, the researchers have decided to examine the effectiveness of the blended learning approach for medical teaching "Congenital adrenal hyperplasia and review of Ambiguous Genitalia" to pediatric physicians. In this research, the following questions were assessed and analyzed: (i) What is the difference between the levels of knowledge about "Congenital adrenal hyperplasia and review of Ambiguous Genitalia" among the study samples before and after blended learning? (ii) What is the attitude of the study samples towards the blended learning method? (iii) What is the relationship between having access to a computer and the level of attitude and perception of the study samples about blended learning?

## Materials and Methods

This research was performed through the interventional study approach. The research samples consisted all postgraduate and undergraduate physicist students of medical training courses who had participated in Congenital Adrenal Hyperplasia and Ambiguous Genitalia class session. Samples were taken gradually during a 6-month period. The data collection tool was a questionnaire that the research centers must complete. The content validity of this questionnaire was examined by knowledgeable professors. In order to assess the reliability of this questionnaire, the retest method and Cronbach's Alpha coefficient was used (87%). All participants who attended this class session were informed about the research and its stages. All participants, who were interested in taking part in this research, filled the pretest questionnaire and received an educational multimedia compact disk. One week later, a class problem solving session was held. The research subjects filled the post-test questionnaire in the next 2-4 weeks. This questionnaire was prepared to examine the level of knowledge, attitude and satisfaction toward blended learning approach. Data analysis was done with the SPSS statistical software (Version 15, Chicago, IL, USA). Data analysis was obtained through descriptive statistics, mean and standard deviation, paired t-test for comparing pretest and post-test scores, variance analysis for mean analysis and Pearson's correlation coefficient for evaluating the relations. A significance level of <.05 was considered.

## Results

In this research, 130 medical students from different educational levels including 10 students (7.7%), 40 externs (30.8%), 19 interns (14.6%), 38 residents (29.2%), and 6 assistant professors (4.6%) participated. Seventeen participants did not mention their educational level. Seventy four of the research participants (56.9%) were women, 44 of them (33.8%) were men and also 12 of participants (9.2%) did not mention their sex. Regarding their employment status, 66.9% were unemployed, 4.6% had an independent and nongovernmental job, 6.2% were contractual employees and 4.6% were conventional employees. Regarding accessibility to a computer, 45.4% of the participants had access to a computer at home, 4.6% at work, 20% at an internet cafe and 26.9% had access from more than one place. The first specific purpose of this research was the comparison of the participants' knowledge toward "Congenital adrenal hyperplasia and ambiguous genitalia" before and after blended learning. Therefore, the research samples filled the pretest questionnaires before learning and 2-4 weeks after learning. It should be mentioned that the scores were out of 10. The results showed that in all groups, there was a meaningful relationship between pretest and post-test scores and the post-test scores were significantly more than the pretest (p<0.01) (Table 1).

**Table 1:** Mean and standard deviation of the pretest and post-test scores in educational levels (scores are out of 10)

**Educational level**	**No.**	**Pre-test Mean±SD**	**Post–test Mean±SD**	***P *value**
Student	10	4.2±1.4	7±3.1	0.008
Extern	40	4.2±1.9	8.6±1.6	<0.001
Intern	19	4.4± 1.5	8.5±2.2	<0.001
Resident	38	4.9±1.9	6.9±2.4	0.050
Assistant professor	6	8±1.1	10±1.1	0.003

Also, the differences between educational groups were investigated. ANOVA showed significant differences between students with Interns (p<0.001), between residents and students (p=0.290) and between interns and residents (p=0.003). The second specific goal of this research was to determine the attitude of the participants towards blended learning. The attitude measurement was compared among different groups. The results showed that the mean attitude level was the highest in assistant professors (84) and the lowest in students (60.7).Variance analysis showed a meaningful relationship between the educational levels and the participants' level of attitude and satisfaction (p=0.007) (Table 2).

**Table 2:** The relationship between educational level and attitude level

**Educational level**	**Number of students**	**Mean attitude and satisfaction score**	**Standard deviation**
Student	10	69.6	7.4
Extern	40	77.05	6.9
Intern	19	75.7	10.3
Resident	38	75.7	6.8
Assistant professor	6	84.0	3.9
Total No.	113	76.0	7.8

As the results showed, those who had higher pretest scores, a better attitude toward the blended learning approach was noticed. Regarding the effect of education on attitude and to determine merely the relationship between the age and satisfaction, the effect of the educational level was omitted. Pearson's correlation coefficient indicated to a significant and positive relationship between the age and satisfaction. As one grows older, the satisfaction score increased (r=0.32, p<0.001).

The other goal of the research was to specify the relationship between accessibility to a computer and the participants' level of attitude towards blended learning approach. Results showed that there was no significant relationship between pretest and post-test scores regarding the research samples’ accessibility to the computer, but there was a significant relationship between the attitude measurement toward the blended learning approach and the participants' accessibility to the computer. Consequently, those who only had access to a computer at work had the lowest attitude and those who had access to a computer at home had the highest attitude toward the blended learning approach (p=0.03).

## Discussion

Generally this research showed that the level of the participants' knowledge towards “Congenital adrenal hyperplasia and review of ambiguous genitalia” had increased significantly after blended learning. There was a meaningful relationship between pretest and post-test scores in all the groups. In such a way that the scores of the post-test, which had been held 2-4 weeks after the education, had increased significantly. The obtained quantities suggest that this educational strategy was an effective method in creating profound and permanent learning of subject matters. Other consistent studies in other countries confirm the results of this study.[6][7][8][9][10]

The learners’ satisfaction is one of the factors which make educational programs effective and successful.[11][12] The results showed that there was a significant relationship between the educational level and the level of the participants' attitude. In such a way that by a raise in the participants' educational level, the level of attitude increased and education contained a significant effect on the attitude of the participants' towards blended learning.

The results showed that there was a positive and significant relationship between age and the level of satisfaction, and by an increase of age, the score of the level of satisfaction increased. Blended learning was efficiently capable of creating flexibility regarding the time, place and speed of learning and provided independency for the learner which was consistent with self leadership feature in adults. The results of this research also indicated that there was a significant relationship between the participants' accessibility to the computer and their attitude and level of satisfaction toward the blended learning strategy. In this case, those who only had access to a computer at work had the lowest attitude and those who had access a computer at home had the lowest attitude towards blended learning (p<0.03). Therefore, accessibility to a computer had a significant effect on acceptance a positive attitude towards electronic strategies.[13][14] It is hoped that the results of this research would be a positive step for improving the medical education level and increasing the effectiveness of medical education strategies through new educational approaches such as blended learning.
